# Author Correction: Human ESCRT-III polymers assemble on positively curved membranes and induce helical membrane tube formation

**DOI:** 10.1038/s41467-020-17082-y

**Published:** 2020-06-24

**Authors:** Aurélie Bertin, Nicola de Franceschi, Eugenio de la Mora, Sourav Maity, Maryam Alqabandi, Nolwen Miguet, Aurélie di Cicco, Wouter H. Roos, Stéphanie Mangenot, Winfried Weissenhorn, Patricia Bassereau

**Affiliations:** 1 Laboratoire Physico Chimie Curie, Institut Curie, PSL Research University, CNRS UMR168, 75005 Paris, France; 2 Sorbonne Université, 75005 Paris, France; 3 Univ. Grenoble Alpes, CEA, CNRS, Institut de Biologie Structurale (IBS), 71, avenue des Martyrs, 38000 Grenoble, France; 4 Moleculaire Biofysica, Zernike Instituut, Rijksuniversiteit Groningen, Nijenborgh 4, 9747 AG Groningen, The Netherlands

**Keywords:** Membrane biophysics, Membrane structure and assembly, ESCRT, Cryoelectron microscopy

Correction to: *Nature Communications* 10.1038/s41467-020-16368-5, published online 29 May 2020.

The original version of this Article contained an error in the spelling of the author Sourav Maity, which was incorrectly given as Sourav Maiti. This has now been corrected in both the PDF and HTML versions of the Article.

