# Supplementary material for: Human ESCRT-III polymers assemble on positively curved membranes and induce helical membrane tube formation
Source: Nat Commun. 2020 May 29;11:2663. doi: 10.1038/s41467-020-16368-5 (PMC7260177; doi:10.1038/s41467-020-16368-5)
Supplement: Supplementary file 1 — Supplementary Information [file 41467_2020_16368_MOESM1_ESM.pdf]

Supplementary Information

Human ESCRT-III Polymers
Assemble on Positively Curved Membranes
and Induce Helical Membrane Tube Formation

Bertin et al,

Supplementary Figures

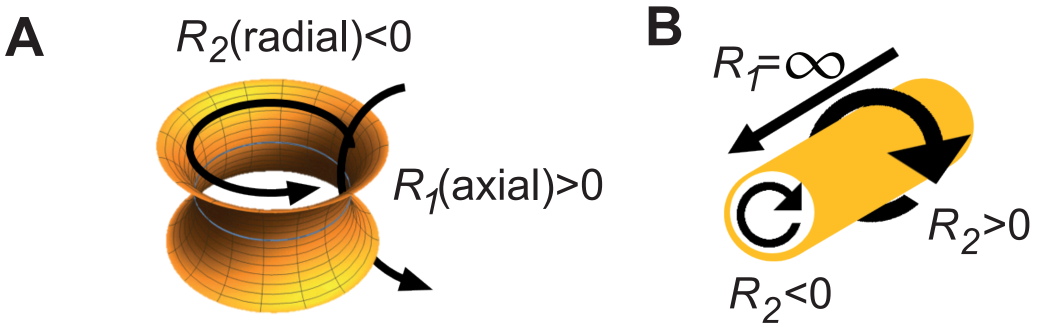

Supplementary Figure 1: Curvatures corresponding to different membrane geometries relevant for ESCRT binding.
The bud necks where ESCRT-III generally assemble are surfaces generally described by 2 principal curvatures that have opposite signs, thus a negative Gaussian curvature $K = \frac{1}{R_1} \times \frac{1}{R_2}$. A neck shape can be close to a catenoid surface (**A**) when the mean curvature $C = \frac{1}{R_1} + \frac{1}{R_2} = 0$, but during the fission processes, C and K can vary. For a comparison, in a tubular structure, the curvature along the axis is null (C_1=0 or equivalently R_1=∞), thus K=0, whereas in the perpendicular direction, the curvature is positive outside of the tube (thus the mean curvature C>0) and negative inside (C<0) (**B**).

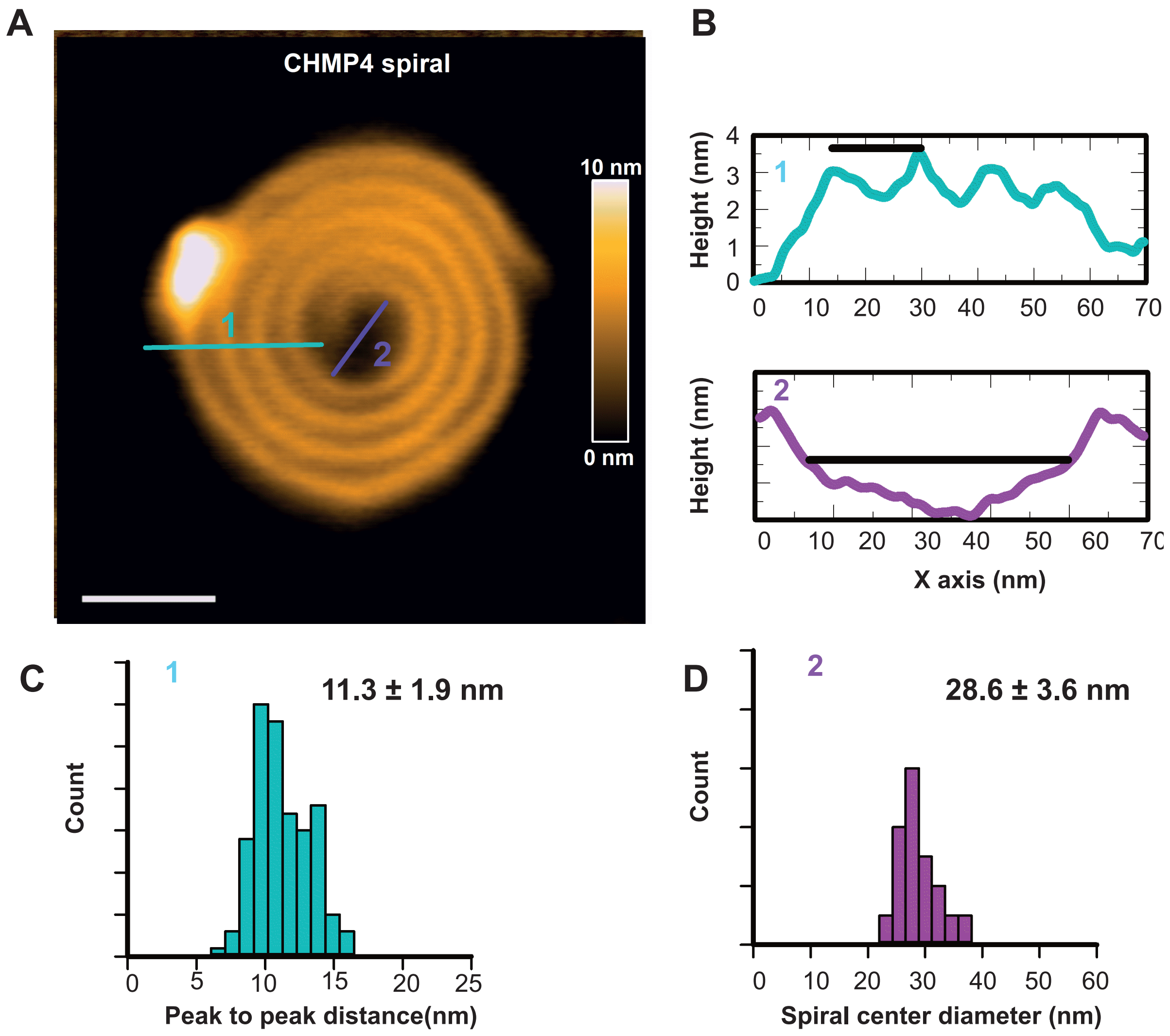

Supplementary Figure 2: Characterization of the CHMP4B-ΔC filaments on supported lipid bilayer with HS-AFM.

A: A typical example of an HS-AFM image of a CHMP4B-ΔC spiral on a supported lipid bilayer.

B: Cross section along the color-coded lines in panel (A).

C and **D**: Histograms of the measured distances as indicated (as an example) by black lines in panel (B). In (**C**): distribution of the inter-filament peak to peak distances (N=134). In (**D**): distribution of central diameters of the CHMP4B spirals (N=18). The mean values ± SD are provided. Source data are provided as a Source Data file.

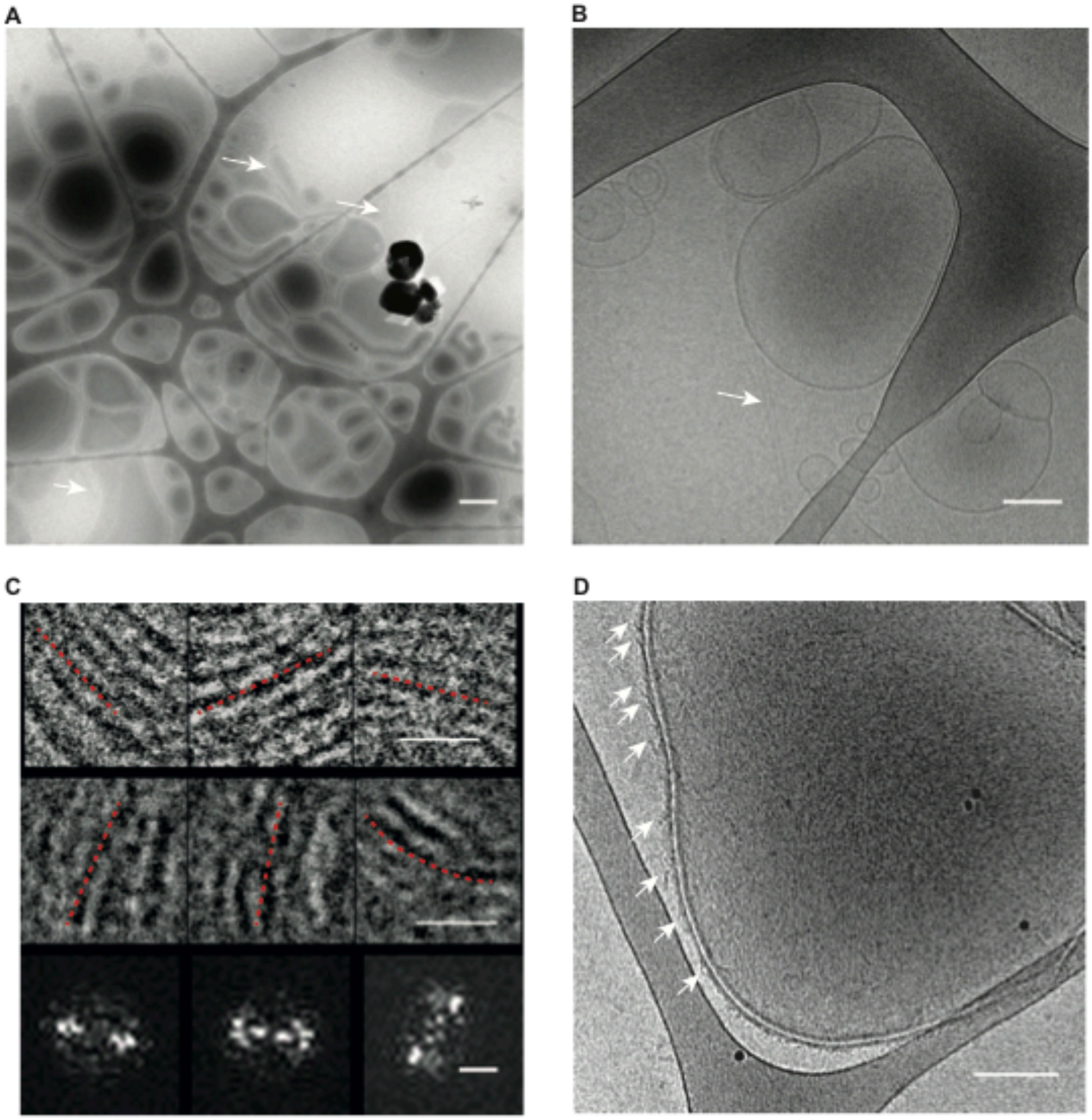

Supplementary Figure 3: Cryo-EM control experiments in the absence of proteins and in the presence of CHMP4B-ΔC or CHMP2A-ΔC/CHMP3.

A and **B**: LUVs resuspended from a lipid dry film and imaged by Cryo-EM at low (**A**) and high (**B**) magnification. Scale bars: A, 250 nm; B, 50 nm. The vesicles are spherical, and approximately 15% of the samples display tubes (white arrows).

C: 2D analysis of lipid tubes in the presence of CHMP4B-ΔC. Scale bars: 20 nm.

Upper row: Boxed tube sections picked from the raw images.

Middle row: Class averages generated by 2D alignment and classification.

Lower row: Fourier transform corresponding to the classes above displaying diffraction peaks, characteristic of the inter-filament distance within the tube. The red dash line corresponds to the tube axis. Scale bar: 1 nm^{-1}

D: Cryo-EM image of LUVs incubated with CHMP2A-ΔC+CHMP3 at 0.5 µM and 3 µM, respectively. Scale bar: 100 nm. CHMP2A-ΔC/CHMP3 form short tubular structures (white arrows) extending out of the vesicle.

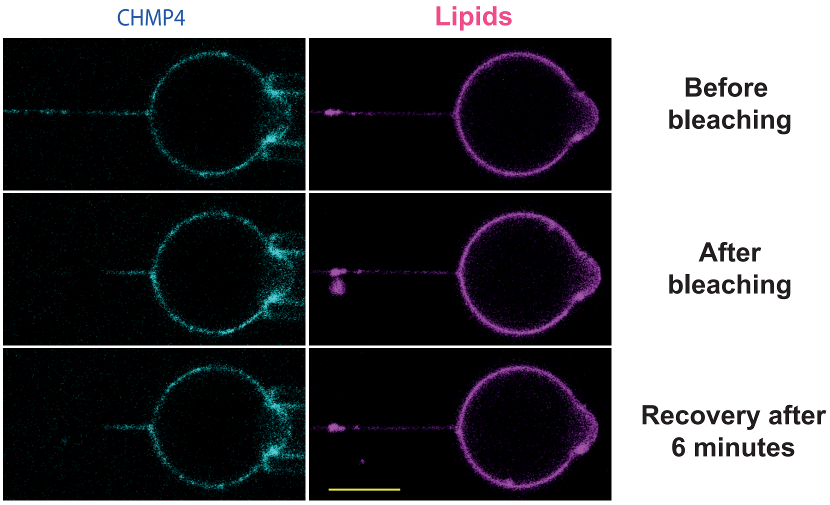

Supplementary Figure 4: CHMP4B-ΔC polymerizes on tubes.
No fluorescence recovery of CHMP4B-ΔC is detected 6 min after photobleaching, indicating the formation of stable polymers onto the tube. Scale bar: 20µm.

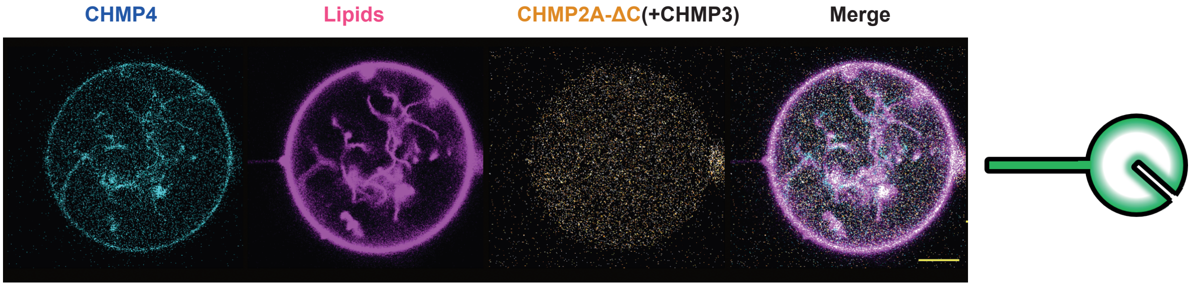

Supplementary Figure 5: CHMP4B-ΔC/CHMP2A-ΔC/CHMP3 binds to positively curved membranes.
Representative confocal images of GUVs in geometry (iv) in the presence of CHMP4B-ΔC and CHMP2A-ΔC+CHMP3 showing a preferential recruitment of CHMP4B-ΔC onto the internal tube. Scale bar: 20µm.

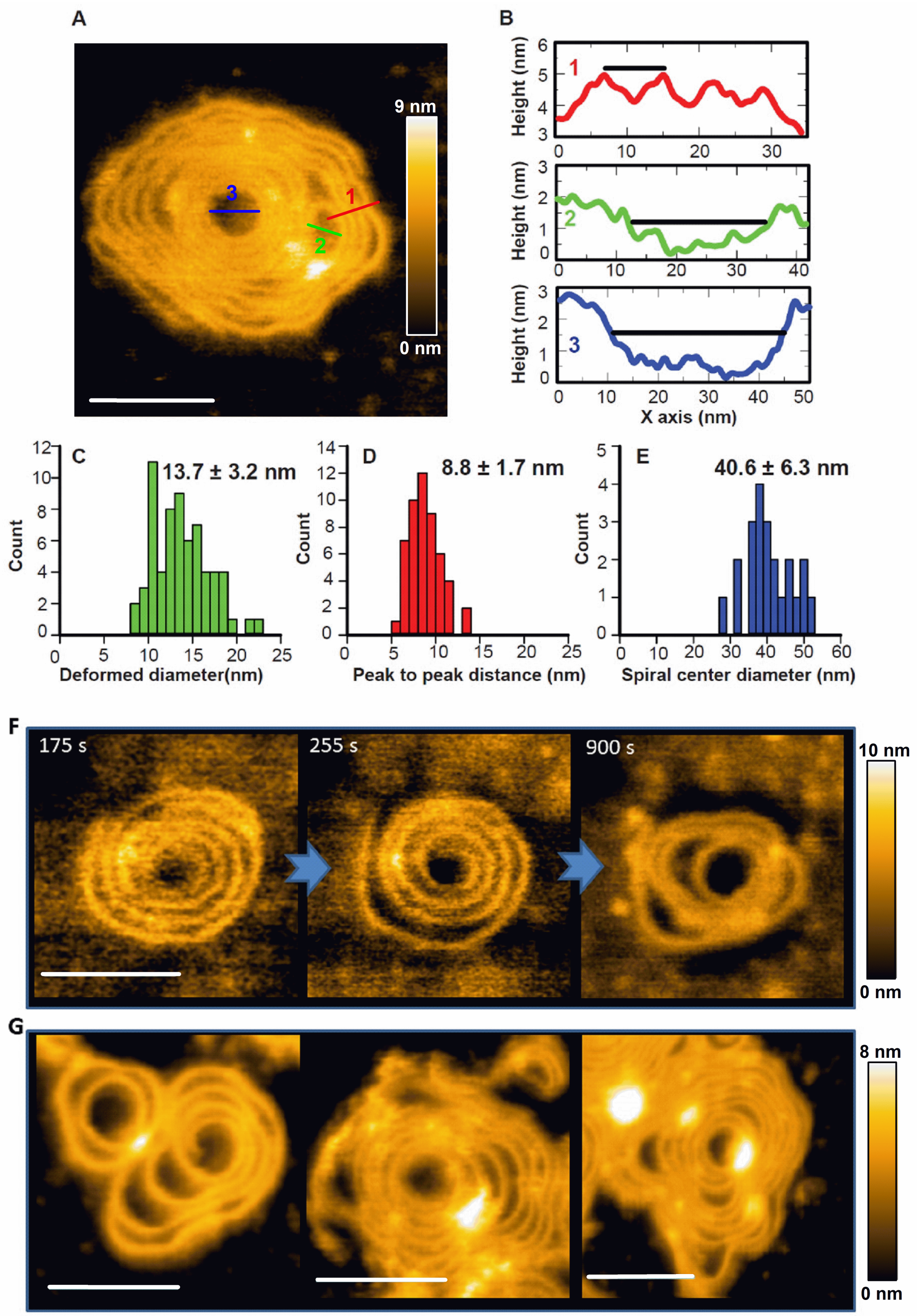

Supplementary Figure 6: Effect of CHMP2B-ΔC on CHMP4B-ΔC spirals, studied by HS-AFM.

A: A typical example of an HS-AFM image of a CHMP4B-ΔC spiral deformed by the addition of CHMP2B-ΔC.

B: Cross-sections along the different colored lines in (A).

C: Distribution of inter-filament distances at the site of deformations (N=65), corresponding to the green line in (B). Source data are provided as a Source Data file.

D: Distribution of inter-filament distances at a non-deformed filament (N=51), corresponding to the red line in (B). Source data are provided as a Source Data file.

E: Distribution of the central diameters of CHMP4B-ΔC spirals deformed by CHMP2B-ΔC (N=22), corresponding to the blue line in (B). Source data are provided as a Source Data file.

The mean values ± SD are provided.

F: Snapshots from Supplementary Movie S4, showing a progressive deformation of the CHMP4B spiral after addition of CHMP2B-ΔC. Scale bar: 100 nm.

G: Images of deformed spirals after being incubated for 15 minutes in 2 μM CHMP2B-ΔC. Scale bars: 100 nm.

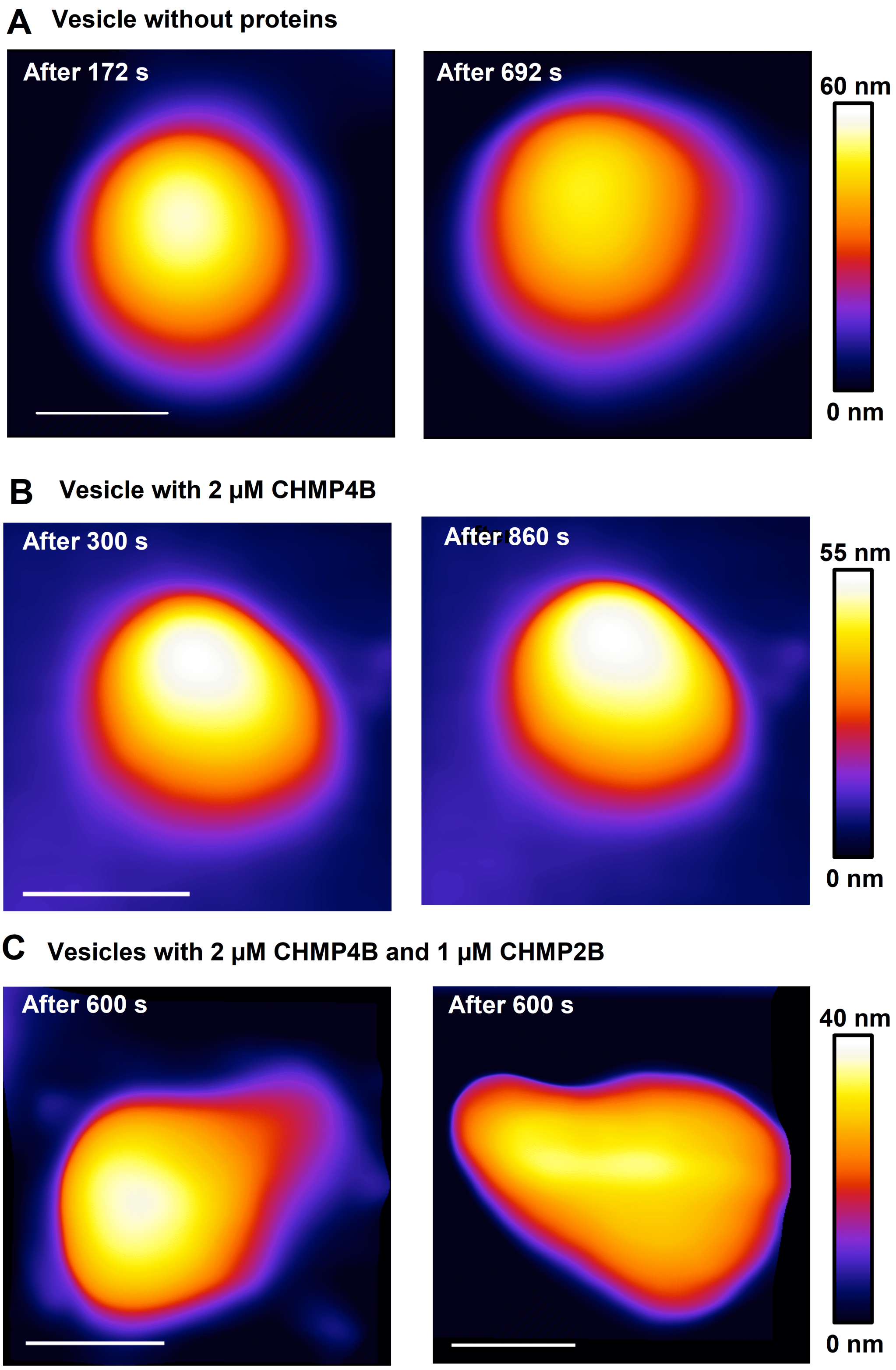

Supplementary Figure 7: Effect of CHMP4B-ΔC and of CHMP2B-ΔC on liposomes, studied by HS-AFM

A: Snapshots of a small liposome in the absence of proteins, imaged by HS-AFM at 2 different time points. Scale bar: 100 nm.

B: Snapshots of a small liposome incubated with 2 µM CHMP4B-ΔC, imaged by HS-AFM at 2 different time points. Scale bar: 100 nm.

C: Snapshots after 600 s of 2 different liposomes first incubated with 2 µM CHMP4B-ΔC, and next with 1 µM CHMP2B-ΔC. Scale bar: 100 nm.

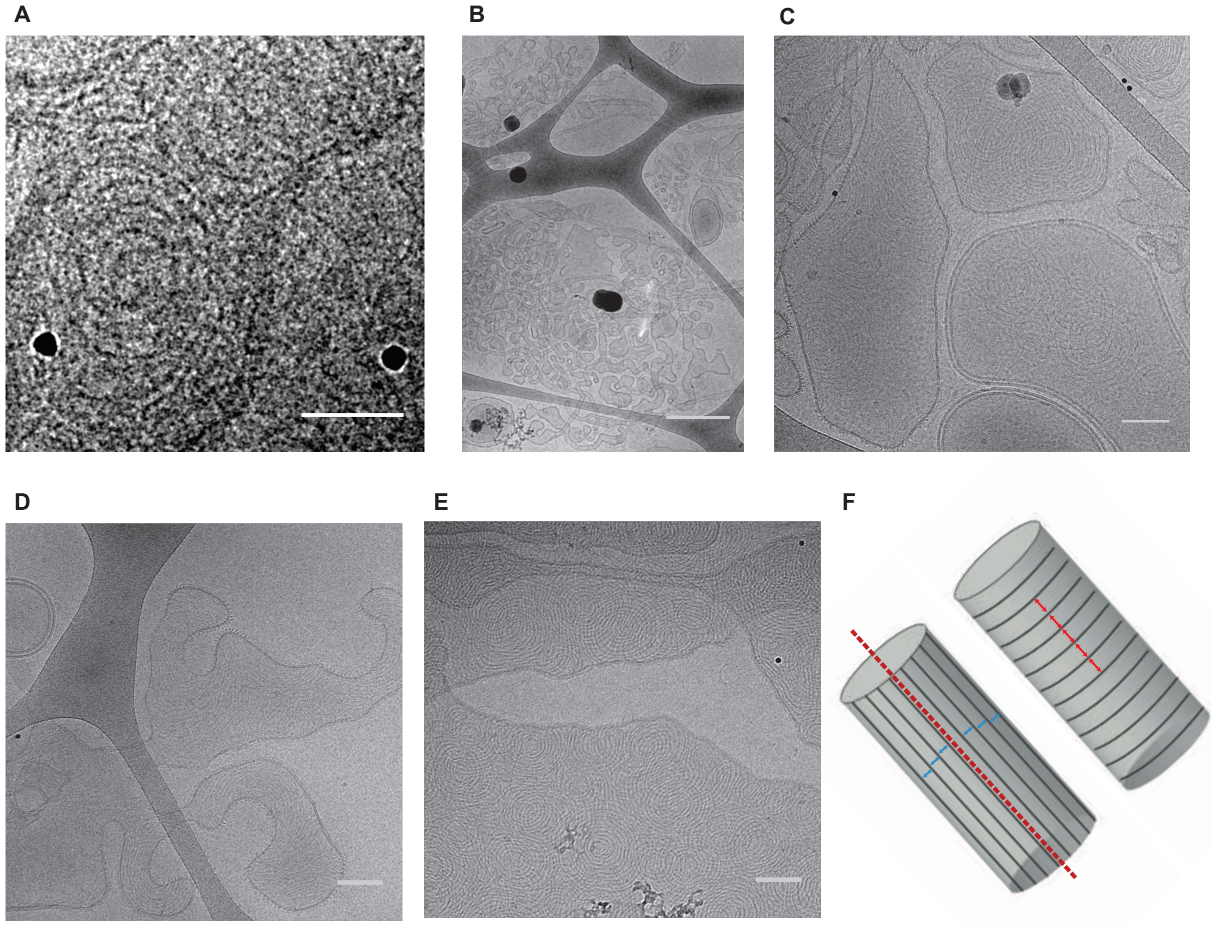

Supplementary Figure 8: Liposome shape changes induced by combinations of CHMP4B-ΔC, CHMP2B-ΔC and CHMP2A-ΔC/CHMP3, studied by Cryo-EM.

A: Cryo-EM images of LUVs incubated with CHMP4B-ΔC and then CHMP2B-ΔC. The spiral of CHMP4-ΔC is deformed by the presence of CHMP2B-ΔC. Scale bar: 100 nm.

B: Low magnification cryo-EM images of LUVs incubated with CHMP4B-ΔC followed by CHMP2A-ΔC and CHMP3. Scale bar: 500 nm.

C-E: Cryo-EM images of LUVs incubated with CHMP4B-ΔC and CHMP2B-ΔC added simultaneously (**C**), CHMP2B-ΔC first and then CHMP4-ΔC (**D**) and CHMP2A-ΔC +CHMP3 and then CHMP4B-ΔC (**E**). Scale Bars: 100 nm.

F: Scheme of the repeat distance along the tube diameter (red arrows) and perpendicular to the tube diameter (blue arrows). Illustration of the axes shown in the FT in Figure 3N.

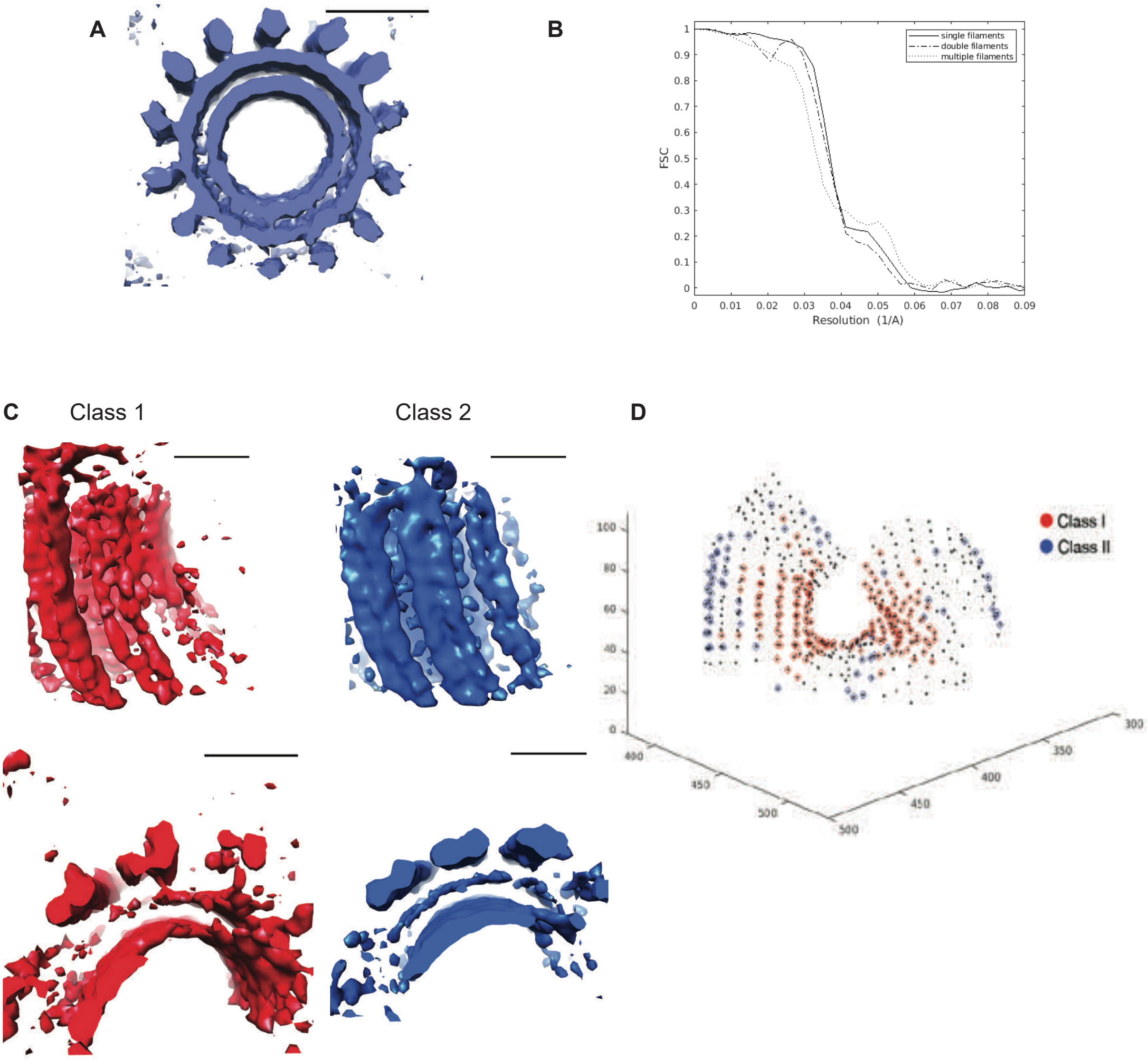

Supplementary Figure 9: Subtomogram averaging of CHMP4B-ΔC/CHMP2B-ΔC on pipe surfaces.

A: Average from boxing out the whole section of a tube using the data corresponding to single filaments (Fig. 4A).

B: Fourier shell correlation for the three reconstructions displayed in Figure 4 (Single filaments: hard line, doublets of filaments: dotted dashed line and network of filaments: dotted line). The resolution was determined from FSC=0.5.

C: Classes (1: red and 2: blue) from MRA classification using the data corresponding to paired filaments. Top: top views of tubes, Bottom: side views of tubes.

D: Spatial distribution on a tube of class 1 (red) and class 2 (blue) resulting from MRA classification. Class 1 particles localize onto negative curvatures while class 2 particles localize onto positively curved portion of the tubes.

Scale bars: 10 nm.

Supplementary Tables

Class number	Repeat distance perpendicular to the tube axis (nm)	Repeat distance parallel to the tube (nm)
1	3.12	3.59
2	2.83	3.1
3	3.54	3.51

Supplementary Table 1: Summary of the repeat distances on helical tubes obtained from cryoET class averaging and Fourier Transform
Distances between parallel filaments along the tube axis and repeat distances between structures perpendicular to the axis are provided for the 3 classes exhibiting regular structures in the 2 perpendicular directions.

Buffer Composition (mM)	Encapsulated Charged GUV	4B encapsulation Non-charged GUV	External
NaCl	10	0	85
Tris	25	25	25
Sucrose	250	100	
Glucose			100

Supplementary Table 2: **Buffers used for CHMP4B-ΔC fusion experiments**

Buffer Composition (mM)	Encapsulated Charged GUV	2A+3 encapsulation Non-charged GUV	External
NaCl	25	50	50
Tris	25	25	50
Sucrose	175	100	
Glucose			100

Supplementary Table 3: Buffers used for CHMP2A-ΔC+CHMP3 fusion experiments

Buffer Composition (mM)	Encapsulated Charged GUV	4B+2A+3encapsulation Non-charged GUV	External
NaCl	10	0	85
Tris	25	25	25
Sucrose	250	100	
Glucose			100

Supplementary Table 4: Buffers used for CHMP4B-ΔC+CHMP2A-ΔC+CHMP3 fusion experiments

Buffer Composition (mM)	Encapsulated Charged GUV	4B+2B encapsulation Non-charged GUV	External
NaCl	10	0	85
Tris	25	25	25
Sucrose	250	100	
Glucose			100

Supplementary Table 5: **Buffers used for CHMP4B-ΔC+CHMP2B-ΔC fusion experiments**

Protein or protein combination	Final protein concentration (after fusion)
CHMP4B	1µM
CHMP2A+CHMP3	1µM+1µM
CHMP2B	1µM
CHMP4B+CHMP2A+CHMP3	0.8µM+0.4µM+0.4µM
CHMP4B+CHMP2B	0.8µM+0.8µM

Supplementary Table 6: Final proteins concentration in the GUVs after fusion

Supplementary Movies Legends

Supplementary Movie 1. CHMP4B spiral oligomerized on a flat SLB, imaged by HS-AFM. HS-AFM imaging of a CHMP4B spiral at a 1 frame/s rate. No observable change in the spiral topography was observed during imaging.

Supplementary Movies 2A-B. 3D reconstructions of two different bare liposomes (segmented in yellow) by cryo-electron tomography. Scale bar: 50 nm.

Supplementary Movie 3. CHMP4B bound to a LUV, analyzed by cryoET (corresponding to Fig. 1C)**.**
Successive orthoslices within a typical cryo-tomogram are visualized. In the segmentation, the membranes are displayed in yellow, free CHMP4B filaments are segmented in blue, while bound CHMP4B spirals are segmented in red. Scale bar: 200 nm.

Supplementary Movie 4. Effect of CHMP2B-ΔC on a CHMP4B spiral, as captured by HS-AFM imaging.
HS-AFM imaging at 1 frame/s of a preformed CHMP4B spiral in the presence of 1 μM CHMP2B-ΔC. It is observable that the spiral loses its structural regularity upon interaction with CHMP2B-ΔC.

Supplementary Movies 5A-B. Combined effect of CHMP4B and CHMP2B-ΔC on the shape of a SUV, as captured by HS-AFM (2 different examples)**.**
The small liposome was imaged after incubation for 10 minutes with 1 μM CHMP2B-ΔC. Initially, there was no observable change in the physical dimension of the liposome. However, a deformation was observed after application of 1μM CHMP2B-ΔC. The images were captured at a 1 frame/s rate.

Supplementary Movie 6 (corresponding to Fig. 4A).
Single ESCRT individual filaments bound to lipid tubes. Inner tube diameter: 14.8 nm, outer tube diameter: 21.7 nm. Protein density diameter: 30.2 nm. Reconstruction from 1721 particles. Frame width: 34 nm.

Supplementary Movie 7 (corresponding to Supplementary Fig. 9A).
Single ESCRT individual filaments bound to lipid tubes. Inner tube diameter: 14.8 nm, ou. Single ESCRT individual filaments bound to lipid tubes visualized onto the whole section of a tube. Frame width: 46.6 nm.

Supplementary Movie 8 (corresponding to Fig. 4B).
Paired ESCRT filaments bound to lipid tubes. Inner tube diameter: 15.4 nm, outer tube diameter: 21.7 nm. Reconstruction from 524 particles. Frame width: 34 nm.

Supplementary Movie 9 (corresponding to Fig. 4C).
High density of filaments bound to lipid tubes. Inner tube diameter: 18.5 nm, outer tube diameter: 25.4 nm. Reconstruction from 381 particles. Frame width: 34 nm.
