# Supplementary material for: Human ESCRT-III polymers assemble on positively curved membranes and induce helical membrane tube formation
Source: Nat Commun. 2020 May 29;11:2663. doi: 10.1038/s41467-020-16368-5 (PMC7260177; doi:10.1038/s41467-020-16368-5)
Supplement: Supplementary file 3 — Description of Additional Supplementary Files [file 41467_2020_16368_MOESM3_ESM.docx]

**Descriptions of Additional Supplementary Files**

**File name:** Supplementary Movie 1

**Description:** CHMP4B spiral oligomerized on a flat SLB, imaged by HS-AFM.

HS-AFM imaging of a CHMP4B spiral at a 1 frame/s rate. No observable change in the spiral topography was observed during imaging.

**File name: Supplementary Movies 2A-B**.

**Description:** 3D reconstructions of two different bare liposomes (segmented in yellow) by cryo-electron tomography. Scale bar: 50 nm.

**File name: Supplementary Movie 3**

**Description:** CHMP4B bound to a LUV, analyzed by cryoET (corresponding to Fig. 1C)**.**

Successive orthoslices within a typical cryo-tomogram are visualized. In the segmentation, the membranes are displayed in yellow, free CHMP4B filaments are segmented in blue, while bound CHMP4B spirals are segmented in red. Scale bar: 200 nm.

**File name: Supplementary Movie 4**

**Description: Effect of CHMP2B-ΔC on a CHMP4B spiral, as captured by HS-AFM imaging.**

HS-AFM imaging at 1 frame/s of a preformed CHMP4B spiral in the presence of 1 µM CHMP2B-ΔC. It is observable that the spiral loses its structural regularity upon interaction with CHMP2B-ΔC.

**File name: Supplementary Movies 5A-B**

**Description:** Combined effect of CHMP4B and CHMP2B-ΔC on the shape of a SUV, as captured by HS-AFM (2 different examples)**.**

The small liposome was imaged after incubation for 10 minutes with 1 µM CHMP2B-ΔC. Initially, there was no observable change in the physical dimension of the liposome. However, a deformation was observed after application of 1µM CHMP2B-ΔC. The images were captured at a 1 frame/s rate.

**File name: Supplementary Movie 6** (corresponding to Fig. 4A).

**Description:** Single ESCRT individual filaments bound to lipid tubes. Inner tube diameter: 14.8 nm, outer tube diameter: 21.7 nm. Protein density diameter: 30.2 nm. Reconstruction from 1721 particles. Frame width: 34 nm.

**File name: Supplementary Movie 7 (**corresponding to Supplementary Fig. 9A).

**Description:** Single ESCRT individual filaments bound to lipid tubes. Inner tube diameter: 14.8 nm, ou. Single ESCRT individual filaments bound to lipid tubes visualized onto the whole section of a tube. Frame width: 46.6 nm.

**File name: Supplementary Movie 8** (corresponding to Fig. 4B).

**Description:** Paired ESCRT filaments bound to lipid tubes. Inner tube diameter: 15.4 nm, outer tube

diameter: 21.7 nm. Reconstruction from 524 particles. Frame width: 34 nm.

**File name: Supplementary Movie 9** (corresponding to Fig. 4C).

**Description:** High density of filaments bound to lipid tubes. Inner tube diameter: 18.5 nm, outer tube

diameter: 25.4 nm. Reconstruction from 381 particles. Frame width: 34 nm.
